# Activation of Inflammatory Networks in the Lungs Caused by Chronic Cold Stress Is Moderately Attenuated by Glucose Supplementation

**DOI:** 10.3390/ijms231810697

**Published:** 2022-09-14

**Authors:** Teng Teng, Hao Yang, Tianqi Xu, Guodong Sun, Xin Song, Guangdong Bai, Baoming Shi

**Affiliations:** Institute of Animal Nutrition, Northeast Agricultural University, Harbin 150030, China

**Keywords:** chronic cold stress, lung, inflammatory network, apoptosis, mitochondrial function, glucose

## Abstract

Mammals that live in cold climates endure months of exposure to low temperature in the winter. The incidence of respiratory diseases has increased. The goal of this study was to investigate the effects of chronic cold stress on lung inflammatory networks, apoptosis, and mitochondrial function via Yorkshire pig models, as well as the ameliorative effect of glucose as energy supplements. Here, two trials were conducted (chronic cold stress and glucose supplementation). The results showed that chronic cold stress induced obvious inflammatory cell infiltration in the lungs and damaged the lung tissue structure. Compared with the Y-Con group, the expression of toll-like receptor 4 (TLR4), myeloid differentiation primary response 88 (MyD88), high mobility group box 1 (HMGB1), nucleotide-binding domain, and leucine-rich repeat protein 3 (NLRP3), IL-1β, IL-2, IL-6, and IFN-γ in the lungs of the Y-CS group was enhanced by chronic cold stress (*p* < 0.05). Moreover, chronic cold stress promoted the expression of the Bax and Mfn2 in lungs of Y-CS group (*p* < 0.05). Interestingly, dietary glucose supplementation significantly reduced inflammatory cell infiltration in the lungs. Moreover, glucose supplementation inhibited the expression of TLR4, MyD88, HMGB1, NLRP3, IL-1β, IL-2, IL-6, IFN-γ, and Bax during chronic cold stress. In conclusion, chronic cold stress promoted inflammatory networks, apoptosis, and mitochondrial fusion in the lungs. Dietary glucose supplementation inhibited the inflammatory network during chronic cold stress.

## 1. Introduction

The northern sites of the globe have more temperate climates in summer, while the environment temperature is extremely low during the winter months in cold climates. The low temperature environment is a thorny problem that people living at high latitudes have to face. They are forced to endure daily temperature below 0 °C for several months. Although most people live in warm environments during the cooler seasons, a significant number of people work in relatively extreme cold environments. There are many studies that have evaluated the negative impacts of low temperature on human health. The cold is known to impair manual performance [1,2], induce cardiovascular disease [3,4], induce peripheral circulation-related disorders [2,5], and cause mental manifestations of depression [6]. Commonly, chronic cold stress causes respiratory disease [7]. Even during a normal winter in the north, up to 29 percent of people still experience cold-like symptoms, such as cough and runny nose [6]. Both long- and short-term exposure to low temperature may lead to inflammatory changes in the airways or worsening of respiratory function [8]. Evidence from animal models suggest that chronic cold stress activates the Nrf2 pathway to induce oxidative stress injury in the lungs [9]. However, few studies have revealed the effects of chronic cold stress on the inflammatory pathways.

Both pathogens and endogenous ligands were recognized by toll-like receptor 4 (TLR4) to initiate inflammatory reactions. TLR4/myeloid differentiation primary response 88 (MyD88) is a classic signaling pathway that involves inflammation [10]. TLR4 is expressed in macrophages and lung bronchial epithelial cells and a primary source of the innate immune system [11]. TLR4 recruits MyD88 to activate nuclear factor kB (NF-kB) to induce inflammatory cytokines, such as IL-6, TNF-α, and IL-1β [12]. In addition, inflammasomes are cytoplasmic high-molecular-weight protein platforms, in response to damage signals [13]. Among inflammasomes, NLRP3 (nucleotide-binding domain and leucine-rich repeat protein 3) has been researched extensively and found to be activated by a spectrum of stimuli widely. Generally, one of the important pathways of NLRP3 activation is TLR4 signaling [14,15]. NLRP3 inflammasome also promotes the release of high mobility group box 1 (HMGB1), which is involved in endotoxemia and sepsis [16]. More interestingly, HMGB1 can also promote apoptosis [17]. Apoptosis is a classical type of programmed cell death. Bax and B-cell lymphoma 2 (Bcl-2) belong to the same family, but their roles are opposite. Bax directly promotes cell apoptosis, contrary to the effect of Bcl2 [18]. Apoptosis culminates in the engulfment and degradation of the apoptotic cell [19]. At present, the interference of chronic cold stress on the inflammatory signaling network and cell death pathway in the lungs is unclear.

Endotherms that are exposed to low temperatures need to expend more energy [20]. Various cellular functions in mammals need ATP for energy supply. Large amounts of ATP are produced by mitochondria to maintain metabolic activities. The normal physiological function of mitochondria depends on the balance of mitochondrial dynamics (fusion and fission) [21]. So far, the regulation of mitochondrial function in the lungs by chronic cold stress is not fully understood. Increasing energy intake is thought to enhance cold adaptation in endothermy animals [22]. Glucose is the preferred energy source for many eukaryotic cells. We will explore whether glucose as energy supplements can alleviate lung injury induced by chronic cold stress.

Pigs are widely used animal models in biomedical research, due to the fact that their physiological and immunological characteristics are similar to those of humans [23,24,25]. This study used Yorkshire pig models to reveal the regulatory mechanism of chronic cold stress on the lung’s inflammatory pathways and apoptosis and mitochondrial functions. Additionally, we also explored the benefits of glucose supplementation on the lungs of pig models during chronic cold stress.

## 2. Results

### 2.1. Chronic Cold Stress Induces Lung Injury and Enhances Inflammatory Factors Expression in Lungs

**Figure 1:**
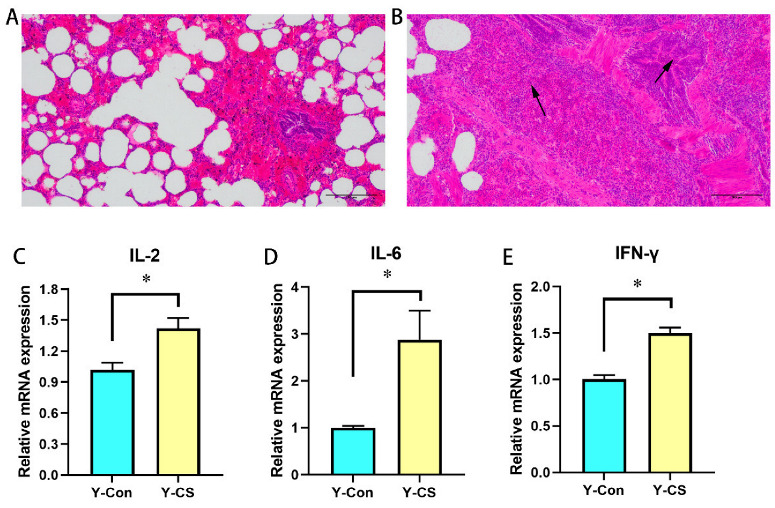
Lung injury assessment and inflammatory cytokines. (**A**) Hematoxylin and eosin (HE) in lung tissue of the control group (Y-Con). (**B**) HE in lung tissue of the cold stress group (Y-CS). Black arrows indicate inflammatory cell infiltration or abnormal development. (**C**) IL-2 mRNA expression in lungs. (**D**) IL-6 mRNA expression in lungs. (**E**) IFN-γ mRNA expression in lungs. Data are expressed as the mean ± SEM, n = 6, * *p* < 0.05.

The pathological sections of the lungs in the first trial are shown in Figure 1. According to the histological observation parameters of the lungs, the morphology of lung tissue in the Y-CS group was observed and compared with that of the Y-Con group as the normal standard. Compared with the Y-Con group (Figure 1A), there were obvious lesions in lung tissue structure of the Y-CS group (Figure 1B). Clear and intact alveoli were rare. Lungs in Y-CS group had a poorly developed bronchial tree with narrow airway lumens. Moreover, alveolar infiltration with eosinophils and intra-alveolar edema were evident in lung tissue of Y-CS group. Chronic cold stress induced obvious lung injury.

The inflammatory cytokines mRNA expression in the lung tissue was assayed. Compared with the Y-Con group, the expression levels of IL-2 (Figure 1C), IL-6 (Figure 1D), and IFN-γ (Figure 1E) in the lungs of the Y-CS group were promoted (*p* < 0.05). Apparently, chronic cold stress overexpresses pro-inflammatory factors in the lungs.

### 2.2. Chronic Cold Stress Activates the TLR4/MyD88 Pathway and Pyroptosis

**Figure 2:**
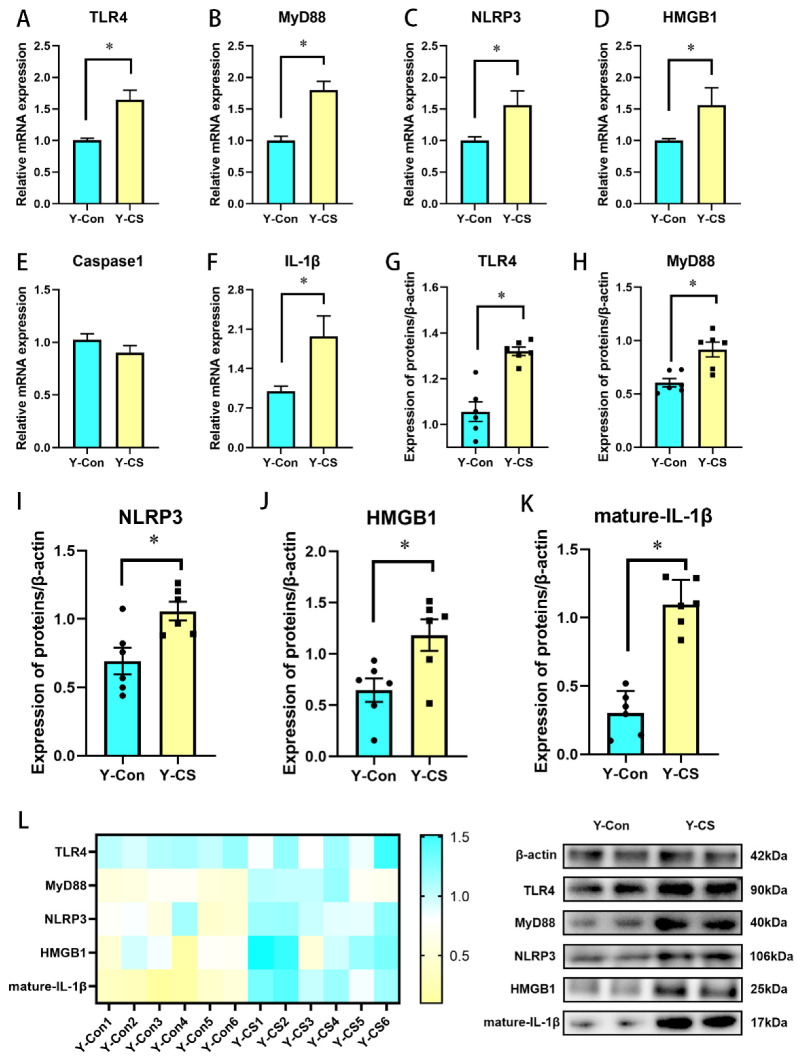
Chronic cold exposure promotes inflammatory pathways in lung tissue. (**A**) Toll-like receptor 4 (TLR4) mRNA expression. (**B**) Myeloid differentiation main response 88 (MyD88) mRNA expression. (**C**) Nucleotide-binding domain and leucine-rich repeat protein 3 (NLRP3) mRNA expression. (**D**) High mobility group box 1 (HMGB1) mRNA expression. (**E**) Caspase1 mRNA expression. (**F**) IL-1β mRNA expression. (**G**) TLR4 protein expression. (**H**) MyD88 protein expression. (**I**) NLRP3 protein expression. (**J**) HMGB1 protein expression. (**K**) Mature-IL-1β protein expression. (**L**) Heat map of protein expression levels. Data are expressed as the mean ± SEM, n = 6, * *p* < 0.05.

Next, we explored changes in the inflammatory pathways and pyroptosis during chronic cold stress. The expression of TLR4 (Figure 2A), MyD88 (Figure 2B), and IL-1β (Figure 2F) was enhanced in lungs of the Y-CS group (*p* < 0.05). In addition, compared with the Y-Con group, NLRP3 (Figure 2C) and HMGB1 (Figure 2D) mRNA expression was increased in lungs of the Y-CS group (*p* < 0.05), although the mRNA expression of Caspase1 was not affected (Figure 2E, *p* > 0.05). We further examined the protein expression levels of these genes (Figure 2L). The protein expression of TLR4, MyD88, NLRP3, HMGB1, and mature-IL-1β in lungs was promoted during chronic cold stress (Figure 2G–K, *p* < 0.05). These data suggested that chronic cold stress activates the TLR4/MyD88 pathway and pyroptosis, thus promoting the release of a large number of pro-inflammatory factors.

### 2.3. Chronic Cold Stress Promotes Apoptosis and Inhibits the Expression of Brain-Derived Nutritional Factors in Lungs

**Figure 3:**
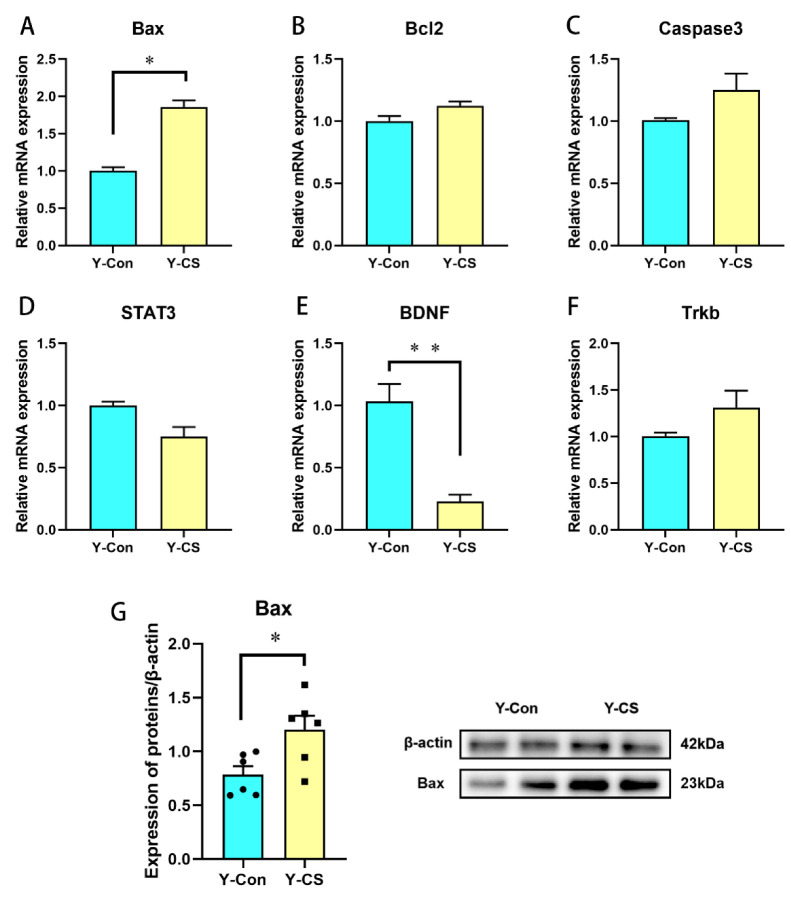
Chronic cold exposure promotes the apoptotic pathway and reduces the expression of brain-derived neural factor in lungs. (**A**) Bax mRNA expression. (**B**) B-cell lymphoma 2 (Bcl2) mRNA expression. (**C**) Caspase3 mRNA expression. (**D**) STAT3 mRNA expression. (**E**) BDNF mRNA expression. (**F**) Neurotrophic receptor tyrosine kinase 2 (Trkb) mRNA expression. (**G**) Bax protein expression. Data are expressed as the mean ± SEM, n = 6, * *p* < 0.05, ** *p* < 0.01.

Then, we focused on the apoptotic pathway under chronic cold stress. Bax mRNA and protein expression were increased in the lungs of the Y-CS group (Figure 3A,G, *p* < 0.05), while Bcl2 and Caspase3 expression were not changed (Figure 3B,C, *p* > 0.05). In addition, the expression of brain-derived nutritional factors (BDNF) was inhibited by chronic cold stress in lungs (Figure 3E, *p* < 0.05). No change in signal transducer, activator of transcription 3 (STAT3), and tropomyosin receptor kinase B (Trkb) mRNA expression was detected (Figure 3D,F, *p* > 0.05). Chronic cold stress promoted apoptosis and inhibits BDNF in lungs.

### 2.4. Chronic Cold Stress Promotes Mitochondrial Fusion in Lungs

**Figure 4:**
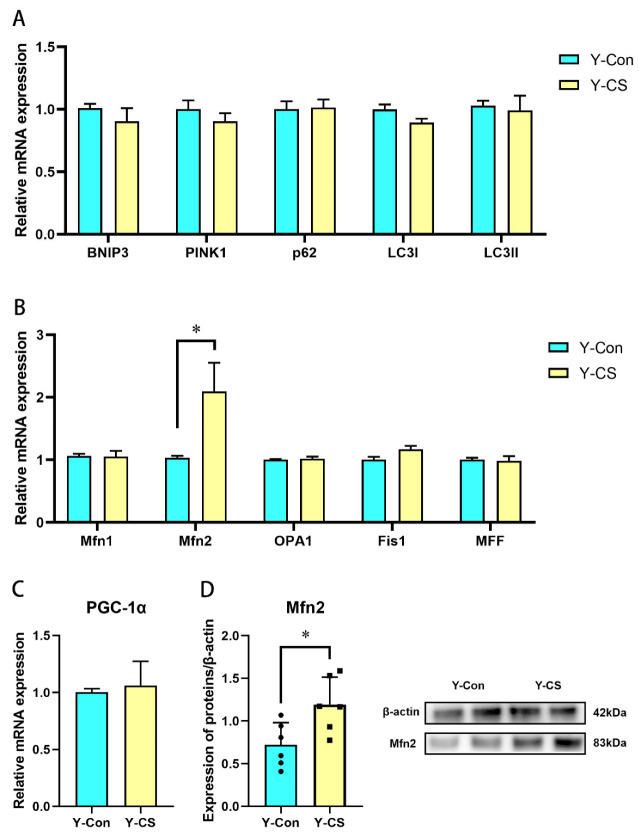
Chronic cold exposure disrupts the mitochondrial kinetic balance in lungs. (**A**) Expression of genes related to mitochondrial autophagy. (**B**) Expression of genes related to mitochondrial dynamic balance. (**C**) PPARG coactivator 1 alpha (PGC-1α) mRNA expression. (**D**) Mitofusin 2 (Mfn2) protein expression. Bax protein expression. Data are expressed as the mean ± SEM, n = 6, * *p* < 0.05.

Energy is used for thermogenesis in response to the cold. The energy supply is extremely dependent on oxidative phosphorylation in the mitochondria. Therefore, we investigated the effects of chronic cold stress on the mitochondrial function in the lungs. In this study, the expression of genes associated with mitochondrial autophagy mitophagy (BNIP3, PINK1, p62, LC3І, and LC3II) in the lungs was not regulated by chronic cold stress (Figure 4A, *p* > 0.05). However, Mfn2 mRNA and protein expression was upregulated (Figure 4B,D, *p* < 0.05), while Mfn1, OPA1, Fis1, and MFF expression was not altered during chronic cold stress (Figure 4B, *p* > 0.05). Incidentally, no significant changes in PGC-1α were observed (Figure 4C, *p* > 0.05). Chronic cold stress enhances mitochondrial fusion in lungs.

### 2.5. Glucose Supplementation Alleviates Lung Injury Induced by Chronic Cold Stress to a Certain Extent

**Figure 5:**
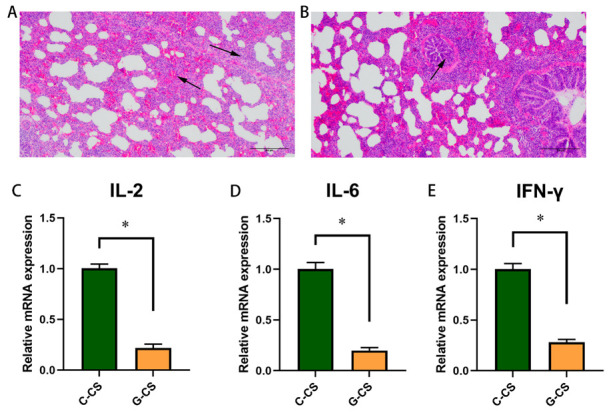
Lung injury assessment and inflammatory cytokines. (**A**) Hematoxylin and eosin (HE) in lung tissue of the cold stress control group (C-CS) group. (**B**) HE in lung tissue of the cold stress with glucose supplementation (G-CS) group. (**C**) IL-2 mRNA expression in lungs. (**D**) IL-6 mRNA expression in lungs. (**E**) IFN-γ mRNA expression in lungs. Data are expressed as the mean ± SEM, n = 6, * *p* < 0.05. Black arrows indicate inflammatory cell infiltration or abnormal development.

In the second trial, the Yorkshire pig models with chronic cold stress was established. In the C-CS group, incomplete alveolar structure and lymphocyte infiltration were observed (Figure 5A). Interestingly, only bronchial stenosis and less inflammatory infiltration were observed in the G-CS group (Figure 5B). The expression of IL-2, IL-6, and IFN-γ mRNA was inhibited in lungs of G-CS group (Figure 5C–E, *p* < 0.05). Thus, glucose supplementation alleviated the lung injury induced by chronic cold stress to a certain extent and inhibited the expression of inflammatory factors.

### 2.6. Glucose Supplementation Inhibits TLR4/MyD88 Pathways and Pyroptosis in Lungs during Chronic Cold Stress

**Figure 6:**
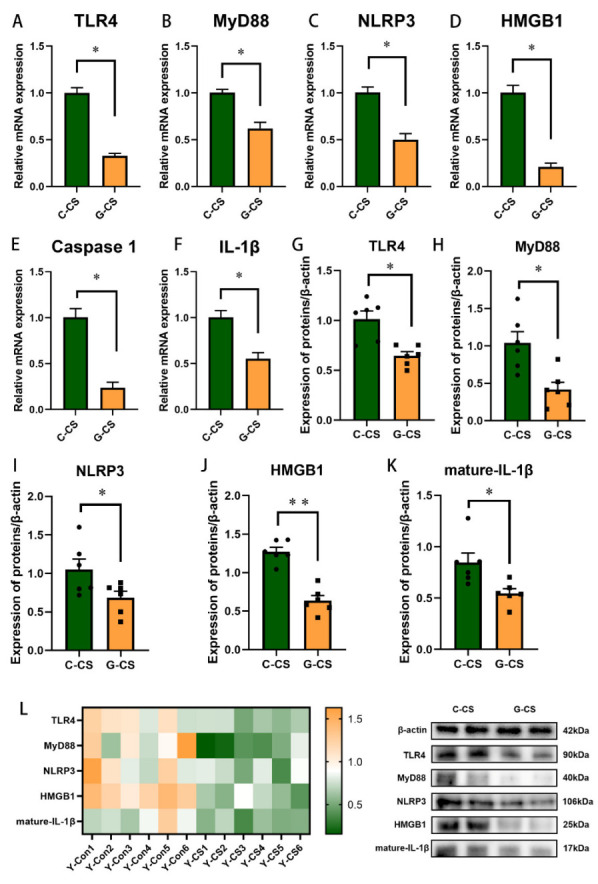
Glucose supplementation improved promotes inflammatory pathways in lung tissue during chronic cold exposure. (**A**) Toll-like receptor 4 (TLR4) mRNA expression. (**B**) Myeloid differentiation main response 88 (MyD88) mRNA expression. (**C**) Nucleotide-binding domain and leucine-rich repeat protein 3 (NLRP3) mRNA expression. (**D**) High mobility group box 1 (HMGB1) mRNA expression. (**E**) Caspase1 mRNA expression. (**F**) IL-1β mRNA expression. (**G**) TLR4 protein expression. (**H**) MyD88 protein expression. (**I**) NLRP3 protein expression. (**J**) HMGB1 protein expression. (**K**) mature-IL-1β protein expression. (**L**) Heat map of protein expression levels. Data are expressed as the mean ± SEM, n = 6, * *p* < 0.05. ** *p* < 0.01.

Based on the mechanism of chronic cold stress inducing inflammation in the lungs in first trial, we focused on the regulation of glucose supplementation on inflammatory pathways and pyroptosis in lungs. The mRNA expression of TLR4, MyD88, NLRP3, HMGB1, Caspase1, and IL-1β was reduced by glucose supplementation in the lungs (Figure 6A–F, *p* < 0.05). Further, we measured their protein expression levels (Figure 6L). The expression of TLR4, MyD88, NLRP3, HMGB1, and mature-IL-1β protein was suppressed in the G-CS group, compared to the C-CS group (Figure 6G–K, *p* < 0.05). Apparently, glucose supplementation suppresses the inflammatory network in lungs during chronic cold stress.

### 2.7. Glucose Supplementation Inhibits Apoptosis Induced by Chronic Cold Stress but Don’t Modulate Mitochondrial Function in Lungs

**Figure 7:**
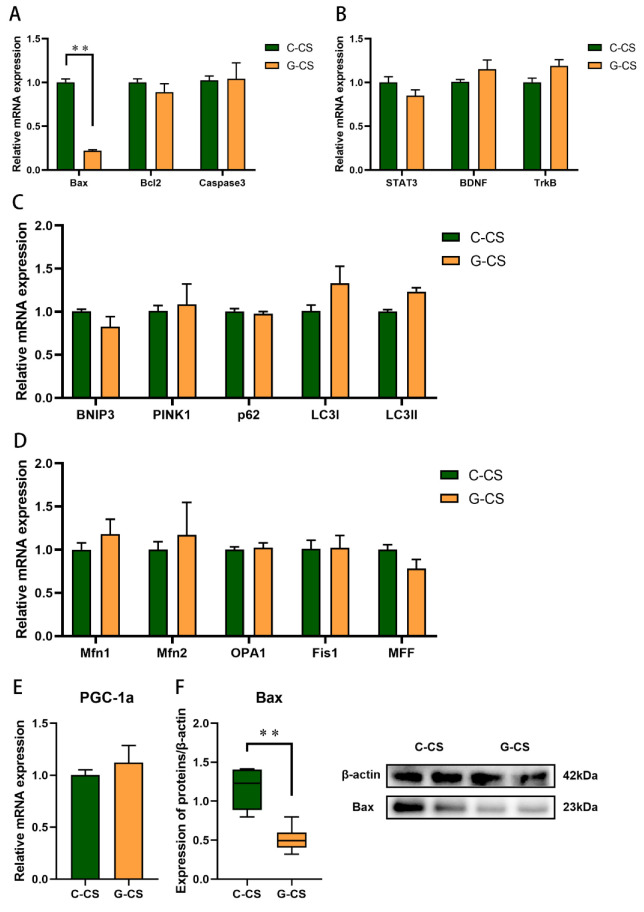
Glucose supplementation improved the apoptotic pathway in lungs during chronic cold exposure. (**A**) Apoptotic pathway related mRNA expression. (**B**) STAT3-BDNF-Trkb pathway mRNA expression. (**C**) Expression of genes related to mitochondrial autophagy. (**D**) Expression of genes related to mitochondrial dynamic balance. (**E**) PGC-1α mRNA expression. (**F**) Bax protein expression. Data are expressed as the mean ± SEM, n = 6, ** *p* < 0.01.

Glucose supplementation inhibited Bax mRNA and protein expression (Figure 7A,F, *p* < 0.05). There was no significant change in the Bcl2 and Caspase3 expression (Figure 7A, *p* > 0.05). In addition, we did not observe significant changes in the expression of genes related to the mitochondrial function (BNIP3, PINK1, p62, LC3І, LC3II, Mfn1, Mfn2, OPA1, Fis1, MFF, and PGC-1α) and STAT3 pathway (Figure 7B–E, *p* > 0.05). These results suggested that glucose supplementation inhibited the apoptosis induced by chronic cold stress.

## 3. Discussion

Living bodies adapt to alterations in their environment by regulating physiological functions. At present, it is widely believed that some diseases are induced when ambient temperature changes overwhelm the capacity of mammals. Changes in ambient temperature are often thought to be an important cause of respiratory diseases [8,26], due to the fact that the respiratory tract maintains a constant interaction with the external environment [27]. The lungs are a vital part of the respiratory system for mammals. Here, we revealed that the mechanisms of lung injury are induced by chronic cold exposure and evaluated the alleviative effect of glucose supplementation through pig models. Inflammatory cell infiltration was evident in the lungs of the Y-CS group under chronic cold stress. Not only that, the alveolar structure was incomplete, and the bronchus was poorly developed. There is no doubt that prolonged exposure to low temperatures can induce lung injury. However, surprisingly, glucose supplementation alleviated the lung injury induced by chronic cold stress to some extent. In G-CS group, histopathological findings showed the inflammatory cell infiltration was reduced. These observations prompted us to focus on the expression of inflammatory cytokines in lungs. We found that the expression levels of IL-1β, IL-2, IL-6, and IFN-γ in the lungs of the Y-CS group were significantly up-regulated, compared with the Y-Con group. In contrast, dietary glucose supplementation down-regulated the expression of these inflammatory factors in the cold-exposed pig models. Generally, natural killer (NK) cells are among the earliest responders to damaged, transformed, or infected host cells through the cytotoxicity and cytokine production. As a proinflammatory factor, IL-2 is produced by Th1 cells [28] and can cooperate with IL-18 to induce extensive IFN-γ release from NK cells [29]. In addition, IL-1β and IL-6 are involved in the inflammatory response. The survival and differentiation of B and T lymphocytes is controlled by IL-6, which contributes to inflammatory processes [30]. Clearly, in our research, chronic cold stress promoted the expression of these inflammatory factors and induced inflammation. Interestingly, glucose supplementation inhibited the expression of proinflammatory factors during chronic cold stress, which was beneficial for mammals.

Based on the high expression of these inflammatory factors, we further analyzed the Toll-like receptor 4 (TLR4) pathway in the lungs of pig models as driven by chronic cold stress. TLR4, a major source of innate immune system [31], is expressed in macrophages and lung bronchial epithelial cells to drive airway inflammation [11]. TLR4 recruits MyD88 at the plasma membrane and induces the oligomerization of MyD88 to form Myddosome [32]. Myddosome activates nuclear factor κB (NF-κB) to release a large number of proinflammatory cytokines, including IL-6, IL-1β, and TNF-α [33,34,35]. Our results showed that chronic cold stress enhanced the expression of TLR4 and MyD88 and induced IL-1β, IL-6, and IFN-γ expression in the lungs. These suggested that chronic cold stress activated the TLR4/MyD88 pathway to induce lung inflammation. Numerous studies have shown that TLR4 activation is associated with NLRP3 inflammasome [36], which is composed of a NLRP3 receptor, Caspase-1, and apoptosis-associated, speck-like protein containing a CARD domain [37,38]. The activated NLRP3 leads to the activation of Caspase-1 and secretion of IL-1β by assembling at the inflammasome [15]. In our study, NLRP3 in the lungs was driven by chronic cold stress. Although Caspase-1 expression was not regulated, mature-IL-1β was secreted in large quantities. In addition, HMGB1 has also been indicated to promote inflammation. HMGB1 not only promoted NLRP3 activation, but it also promoted apoptosis [39,40]. Its high expression also contributed to inflammation during chronic cold stress. Interestingly, dietary glucose as an energy supplement effectively inhibited the TLR4/MyD88 pathway, as well as the NLRP3, Caspase-1, and HMGB1 expression during chronic cold stress. Low temperature environments intensify the energy consumption of endotherms, which require the body to mobilize large amounts of adenosine triphosphate (ATP). TLR4 is involved in promoting the process of glycolysis to rapidly produce ATP, which depends on mitochondrial function [41,42]. However, the high activity of the mitochondria also promotes the release of ROS and oxidative stress damage [43]. Increasing the energy level of the diet may have eased the stress on ATP production during chronic cold stress, thereby inhibiting inflammatory pathways. However, this remains to be confirmed by a larger study.

Apoptosis is the active process of programmed cell death, which is regulated by environmental conditions, in order to maintain homeostasis. In this process, Bax promotes apoptosis and Bcl2 protects cells from death [18]. The ratio of these two proteins determines the fate of apoptosis. Cells are susceptible to apoptosis when Bax is in excess [44]. Apoptosis culminates in the engulfment and degradation of the apoptotic cell [19]. We found that chronic cold stress increased the expression level of Bax in the lungs, indicating that apoptosis was enhanced. Glucose supplementation attenuated the Bax expression in the lungs of cold-exposed pigs, thereby inhibiting apoptosis.

Neurotrophic factors are important growth factors that depend on the nervous system. BDNF is one of the key members of the neurotrophic factors. BDNF coordinates lung smooth muscle formation and innervation by extrinsic neuronal pathways [45]. BDNF expression binds with high affinity to the TrkB [46]. A recent study has shown that BDNF-TrKB signaling can mediate repairs after lung injury by promoting the regeneration of the alveoli [46]. Our data indicated that BDNF expression in the lungs was significantly inhibited by chronic cold stress. Obviously, that is unfavorable for the development of the lungs. In addition, energy supplementation did not restore the expression level of BNDF. This might be one of the important reasons why glucose supplementation did not completely alleviate the lung injury induced by chronic cold stress.

The production ATP of normal cells relies on mitochondrial oxidative phosphorylation primarily. Mitochondria produce ATP through oxidative phosphorylation at the inner mitochondrial membrane. Damaged mitochondrial are inefficient in the ATP, but produce more ROS in cells [47]. Excessive ROS can induce cell damage and death [48]. Commonly, mitochondria are dynamic organelles and exist in a steady state of fusion and fission events. The balance of fusion or fission to either extreme is driven by pathological insults [21]. The mitochondrial fusion is regulated by Mfn1 and Mfn2 for the outer membrane and OPA1 for the inner membrane [49,50], whereas Fis1 and MFF are responsible for mitochondrial fission [21]. The high demand for ATP in mammals exposed to low temperatures forced us to focus on changes in mitochondrial function. We found that the expression of Mfn2 in the lungs was enhanced under chronic cold exposure. This result suggested that mitochondrial fusion was promoted. As a mitochondrial outer membrane protein, Mfn2 is considered to be a key regulator of mitochondrial fusion and mitochondrial metabolism [51]. Its high expression promoted mitochondrial fusion and might impair the physiological function of the mitochondria. Nonetheless, several ideas highlight the beneficial effects of mitochondrial fusion, of which, the promotion of fusion can produce more ATP to accommodate higher energy demands during chronic cold stress [52]. In short, these implied that low temperatures increase the body’s need for ATP, but might cause mitochondrial function to be disrupted. In addition, energy supplementation with glucose did not alter mitochondrial kinetic balance in the lungs of cold-exposed pigs. These results also implied that the main mechanism by which glucose supplementation alleviated chronic cold expose-induced lung injury was not by regulating mitochondrial function, but inhibiting the inflammatory pathways.

## 4. Materials and Methods

### 4.1. Animals, Administrations, and Procedures

In this study, all protocols were approved by the Ethical and Animal Welfare Committee of Heilongjiang Province, China. The proposals and procedures for the care and treatment of animals were approved by the Institutional Animal Care and Use Committee of Northeast Agricultural University (NEAU-[2011]-9).

Firstly, a trial of chronic cold stress was conducted (Exp. 1). A total of 12 Yorkshire pigs (females) were divided into two group: control (Y-Con, 24.83 kg ± 0.64 kg, n = 6) and chronic cold stress (Y-CS, 24.80 kg ± 0.61 kg, 7 ± 3 °C, n = 6) groups. The environment temperature of Y-Con group (17 ± 3 °C) was maintained by electronic heaters (GSM501, Guangzhou Rongce Electronics, Guangzhou, Guangdong, China), and the environment temperature of Y-CS group (7 ± 3 °C) was derived from natural conditions. This trial lasted for 21 days. The diets involved in Exp. 1 were shown in Table 1.

Next, in the second trial, we established cold-exposed Yorkshire pig models with glucose diets (Exp. 2). A total of 12 Yorkshire pigs (females) were randomly divided into control (C-CS, 23.54 ± 0.84 kg, n = 6) and glucose diet (G-CS, 23.76 ± 0.78 kg, n = 6) groups. The environment temperature of cold stress group (under natural conditions) was 8 ± 3 °C. This trial lasted for 22 days. The diets of C-CS group were shown in Table 1, and the diets of G-CS group were shown in Table 2.

All animals in this study were fed separately from a single metabolic cage (including water dispensers). They were provided access to food and water ad libitum during the time of the experiment. Animal cages were cleaned and sanitized daily during these two experiments. The diets involved in this study were formulated (Table 1 and Table 2) to reference the Ministry of Agriculture of the People’ s Republic of China (MOA, 2020) and National Research Council (NRC, 2012) recommended requirement. Nutrient levels in the two diets were calculated as 90% dry matter. The percentage of crude protein was the analytical value.

### 4.2. Sample Collection

All pigs in this research were fasted overnight for 12 h before slaughter. A total of 3 g of lungs was quickly collected and then frozen in liquid nitrogen. Then, these samples were transferred to the −80 °C refrigerator for storage. A total of 1 cm^2^ of lungs was cut and preserved in 10% formaldehyde solution for tissue section observation.

### 4.3. Hematoxylin and Eosin (HE) Staining

HE staining in this research was conducted according to routine protocols. Lung tissues were fixed with 10% formaldehyde solution, embedded in paraffin, and then they were sectioned into thin slices with a microtome (Leica RM2016, Leica, Nussloch, Germany) and laced with hematoxylin and eosin. Stained lung tissue sections were viewed with a Nikon Eclipse Ci-L microscope (Nikon, Tokyo, Japan) at 100 × magnification. All images were captured (scale bar = 100 μm) with the Nikon DS-F12 digital camera (Nikon, Tokyo, Japan).

### 4.4. Total RNA Extraction, Reverse Transcription, and Relative Quantitative Real-Time PCR

The total RNA in lung tissue samples was isolated according to the manufacturer’s instructions via TRIzol Reagent (Invitrogen, Carlsbad, CA, USA). The quality of the total RNA was determined by confirming that the ratio of OD_260_ and OD_280_ was between 1.8 and 2.0. The total RNA was reverse-transcribed using a PrimeScript TM RT reagent kit (Takara, Biotechnology, Dalian, China). The total RNA was reverse-transcribed using an integrated first-strand cDNA synthesis kit (Dining, Beijing, China). Next, the 2 × Fast qPCR master mixture (Dining, Beijing, China) was used to perform real-time PCR in an ABI 7500 fast real-time PCR system (Foster City, CA, USA). Every reaction was performed at least 2 times. The relative amount each target mRNA for was normalized to the β-actin mRNA levels. Information on all the primers is shown in Table 3. The relative gene expression was calculated using the 2^−ΔΔCt^.

### 4.5. Western Blot Analysis

Firstly, the total protein in lung tissues was acquired with RIPA buffer mix, including 1% PMSF (Beyotime Biotechnology, Shanghai, China). The protein concentrations were determined by an enhanced BCA protein assay kit (Beyotime Biotechnology, Shanghai, China). After SDS-PAGE, protein was transferred to a polyvinylidene fluoride (PVDF) membrane through electrophoretic transfer. The membrane was blocked in TBST (containing 5% nonfat dry milk) at room temperature for 2 h. Subsequently, the blots were incubated with primary antibodies (TLR4, MyD88, NLRP3, HMGB1, Mature-IL-1β, Bax, and Mfn2) overnight at 4 °C. After thoroughly washing three times by TBST, the membranes were incubated with the second antibody for 2 h at room temperature. After the membranes were washed, antibody reactivity was detected by chemiluminescence through the BeyoECL star fluorescence detection kit (Beyotime Biotechnology, Shanghai, China). These bands were imaged by a gel imaging and analysis system (UVItec, Cambridge, Britain), and band intensity was assessed using the Image J system, with correction for background and loading controls. β-actin was used to normalize the intensity of the bands. All the antibodies information involved in this study is displayed in Table S1.

### 4.6. Statistical Analysis

The normality and homogeneity of variances of the data were evaluated. Then, we analyzed the data by “*t*-Test” (SPSS 22.0; IBM-SPSS Inc., Chicago, IL, USA) and visualized them via GraphPad Prism (Graph Pad Software Inc., San Diego, CA, USA). The data were expressed as the means ± SEM. Differences were considered significant when *p* < 0.05.

## 5. Conclusions

**Figure 8:**
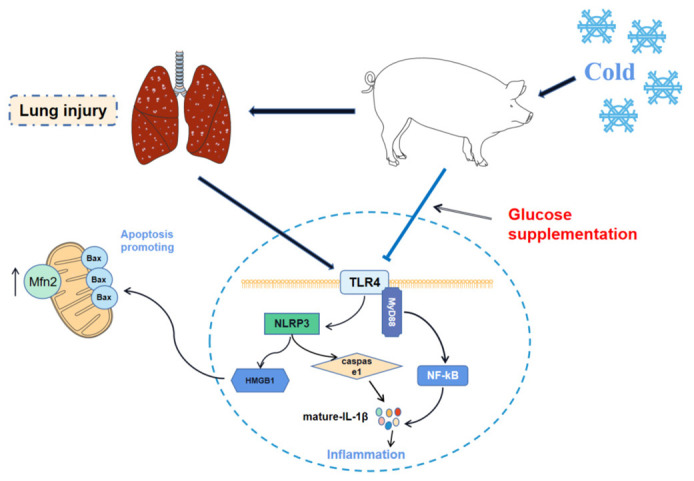
Glucose supplementation alleviates the lung injury induced by chronic cold exposure to some extent. Black arrows indicate up-regulation.

Here, we reveal the mechanism of lung injury caused by chronic cold stress and the alleviating effects of glucose supplementation in pig models. On the one hand, our data illustrated that chronic cold stress induced lung injury by promoting inflammatory pathways, apoptosis, and mitochondrial fusion. On the other hand, dietary glucose supplementation could inhibit the TLR4/MyD88 pathway to some degree to alleviate the lung injury caused by chronic cold stress (Figure 8). This study provides a new theoretical basis and idea to prevent the lung injury induced by chronic cold stress.

## Figures and Tables

**Table 1:** Composition of experimental diets (90% of dry matter).

Basic Diet Ingredients	Content (%)
Corn	73.00
Soybean meal, de-hulled	15.30
Full-fat soybean meal, puffed	5.00
Fish meal	2.00
Soybean oil	1.00
L-Lysine	0.39
DL-Methionine	0.04
L-Threonine	0.12
L-Tryptophan	0.02
Calcium hydrogen phosphate	1.19
Limestone	0.66
Salt	0.28
Premix ^A^	1.00
**Chemical levels ^B^**	
Net energy (Mcal/kg)	2.50
Crude protein	16.05
Lysine	0.98
Methionine	0.29
Threonine	0.60
Leucine	0.17
Calcium	0.66
Total phosphorus	0.56
Available phosphorus	0.33
Sodium	0.14
Chlorine	0.19

^A^ Provided the following per kilogram of diet: Fe, 160 mg; Cu, 150 mg; Mn, 40 mg; Zn, 140 mg; Se, 0.4 mg; I, 0.5 mg; vitamin A, 8000 IU; vitamin D3, 2000 IU; vitamin E, 30 mg; vitamin B1, 1.60 mg; vitamin B2, 5.00 mg; vitamin B6, 5.00 mg; vitamin B12, 0.01 mg; pantothenic acid, 20 mg; niacin, 15 mg; biotin, 0.05 mg. ^B^ Chemical levels were calculated values (the percentage of crude protein is the actual detected value). The proportion of dry matter in the diet is 90%.

**Table 2:** Composition of glucose-supplemented diets (90% of dry matter).

Basic Diet Ingredients	Content (%)
Corn	60.68
Soybean meal, de-hulled	17.53
Full-fat soybean meal, puffed	5.00
Fish meal	2.00
Glucose	10.00
Soybean oil	1.00
L-Lysine	0.35
DL-Methionine	0.05
L-Threonine	0.11
L-Tryptophan	0.01
Calcium hydrogen phosphate	1.25
Limestone	0.62
Salt	0.40
Premix ^A^	1.00
**Chemical levels ^B^**	
Net energy (Mcal/kg)	2.63
Crude protein	16.05
Lysine	0.98
Methionine	0.29
Threonine	0.60
Leucine	0.17
Calcium	0.66
Total phosphorus	0.56
Available phosphorus	0.34
Sodium	0.19
Chlorine	0.26

^A^ Provided the following per kilogram of diet: Fe, 160 mg; Cu, 150 mg; Mn, 40 mg; Zn, 140 mg; Se, 0.4 mg; I, 0.5 mg; vitamin A, 8000 IU; vitamin D3, 2000 IU; vitamin E, 30 mg; vitamin B1, 1.60 mg; vitamin B2, 5.00 mg; vitamin B6, 5.00 mg; vitamin B12, 0.01 mg; pantothenic acid, 20 mg; niacin, 15 mg; biotin, 0.05 mg. ^B^ Chemical levels were calculated values (the percentage of crude protein is the actual detected value). The proportion of dry matter in the diet is 90%.

**Table 3:** The real-time PCR primers.

Gene	GenBank ID	Primer Sequences (5’ to 3’)
β-actin	AY550069	F: ATGCTTCTAGGCGGACTGT
		R: CCATCCAACCGACTGCT
Bax	XM_005664710	F: ATGGAGCTGCAGAGGATGAT
		R: AAAGTAGAAAAGCGCGACCA
Bcl2	NM_001164511.2	F: ACTTCTGCGAAAGCGAATTGCC
		R: AGCCTCCGTTTTGCCTTATCC
Caspase3	NM_214131	F: CGGACAGTGGGACTGAAGTA
		R: GATCCGTCCTTTGAATTTCG
NLRP3	NM_001256770.2	F: CTGGGACTCTGACTAGGGCT
		R: TTTTTCTGTCTGGCCCCGAG
HMGB1	NM_001004034.1	F: GAGGAAACTTGAGACCCACCA
		R: GTGTCCTTCCTTCCCTCATGT
Caspase1	NM_214162.1	F: TACAAGAATCCCAGGCGGTG
		R: CCTTTGGGCTATGTCTGGGG
TLR4	NM_001113039.2	F: CAGTCAAGATACTGGACCTGAGC
		R: GGCTCCCAGGGCTAAAACTCT
MyD88	NM_001099923.1	F: CCATTCGAGATGACCCCCTG
		R: TAGCAATGGACCAGACGCAG
IL-1β	NM_2140551.1	F: GCCAACGTGCAGTCTATGGAGTG
		R: GGTGGAGAGCCTTCAGCATGTG
PGC-1α	NM_213963.2	F: ATGGAGCAATAAAGCGAAGAGCATTTG
		R: GAGGAGGGTCATCATTTGTGGTCAG
Mfn1	NM_001315732.1	F: TGGACTTTATCCGAAACCAGATGAACC
		R: AACCTTATTTGCCACCTCCTCTGTAAC
Mfn2	XM_021095369.1	F: CCACACCACCAACTGCTTCCTG
		R: TCTTGACGCTCCTCTTCTCCTCTG
MFF	NM_001244126.1	F: CAGGTTCCAGAGAGAATTGTCGTAGC
		R: TTAGTGCCAGAGGTTTAAAGGGAGTTG
Fis1	XM_021086263.1	F: CAGACAGAGCCACAGAACAACCAG
		R: CAAGTCCAATGAGTCCAGCCAGTC
OPA1	XM_021070063.1	F: ACAGAGGATGGTGCTTGTTGACTTAC
		R: ACACAGTATGATGGCGTTGGGATTC
STAT3	NM_001044580.1	F: CAGCGGTAAGACCCAGATCC
		R: AGGGTAGAGGTAGACCAGCG
BDNF	NM_214259.2	F: CAGAGCAGCTGCCTTGATGTT
		R: CTTTCATGGGGGCAGCCTTC
TrkB	XM_021064650.1	F: TCTCGGTCTACGCTGTGGTA
		R: GCAGCATCAACCAACAAGCA
BNIP3	XM_003359404.4	F: GAGGAGGATTACATGGAGAGGAGGAG
		R: TCGGGTGCTTGAAGAGGAGGAAC
PINK1	XM_021095478.1	F: GGCGGTGATTGACTACAGCAAGG
		R: TGGTAACTGCGGCTTTCAAGGTG
p62	NM_001244307.1	F: CTGCCTGAAGACTATTACACGAGACC
		R: GAAGATGCTTGTGCCGAGGATAGAG
LC3Ⅰ	NM_001170827.1	F: GCCTTCTTCCTGCTGGTGAACC
		R: GGGAGGCGTAGACCATGTAGAGG
LC3Ⅱ	NM_001190290.1	F: TTCTTCCTGTTAGTGAACGGACATAGC
		R: ATCCATCTTCATCCTTCTCGCTTTCG

## Data Availability

The data presented in this study are available on request from the corresponding author. The data are not publicly available, due to the fact that we are conducting further experiments.

## Supplementary Materials

The following supporting information can be downloaded at: https://www.mdpi.com/article/10.3390/ijms231810697/s1.
